# Functional and Comparative Genomics of *Hoxa2* Gene *cis-*Regulatory Elements: Evidence for Evolutionary Modification of Ancestral Core Element Activity

**DOI:** 10.3390/jdb4020015

**Published:** 2016-03-26

**Authors:** Adam Davis, Michael C. Reubens, Edmund J. Stellwag

**Affiliations:** 1 Department of Biology and Physical Sciences, Gordon State College, Barnesville, GA 30204, USA; 2 The Scripps Research Institute, 10550 N, Torrey Pines Road, MB3, La Jolla, CA 92037, USA; michaelreubens82@gmail.com; 3 Department of Biology, Howell Science Complex, East Carolina University, Greenville, NC 27858, USA; stellwage@ecu.edu

**Keywords:** Japanese medaka, *Hox* PG2 gene expression, *hoxa2a*, *ψhoxa2b*, embryonic development, *cis-*regulatory elements, vertebrate evolution, gene regulation

## Abstract

*Hoxa2* is an evolutionarily conserved developmental regulatory gene that functions to specify rhombomere (r) and pharyngeal arch (PA) identities throughout the Osteichthyes. Japanese medaka (*Oryzias latipes*) *hoxa2a,* like orthologous *Hoxa2* genes from other osteichthyans, is expressed during embryogenesis in r2–7 and PA2-7, whereas the paralogous medaka pseudogene, *ψhoxa2b*, is expressed in noncanonical *Hoxa2* domains, including the pectoral fin buds. To understand the evolution of *cis*-regulatory element (CRE) control of gene expression, we conducted eGFP reporter gene expression studies with extensive functional mapping of several conserved CREs upstream of medaka *hoxa2a* and *ψhoxa2b* in transient and stable-line transgenic medaka embryos. The CREs tested were previously shown to contribute to directing mouse *Hoxa2* gene expression in r3, r5, and PA2-4. Our results reveal the presence of sequence elements embedded in the medaka *hoxa2a* and *ψhoxa2b* upstream enhancer regions (UERs) that mediate expression in r4 and the PAs (*hoxa2a* r4/CNCC element) or in r3–7 and the PAs *ψhoxa2b* r3–7/CNCC element), respectively. Further, these elements were shown to be highly conserved among osteichthyans, which suggests that the r4 specifying element embedded in the UER of *Hoxa2* is a deeply rooted rhombomere specifying element and the activity of this element has been modified by the evolution of flanking sequences that redirect its activity to alternative developmental compartments.

## 1. Introduction

Clustered *Hox* genes are a family of evolutionarily-related developmental regulatory genes that function to pattern regional tissue identities along the anterior-posterior (A-P) axis of animal species [1]. *Hox* clusters comprise up to 14 genes, which are expressed along the A-P axis during embryonic development collinear with their physical location within a cluster [2,3,4]. Multiple genome level duplications have expanded the total number of *Hox* clusters from one in chordates to four in tetrapods and at least seven or eight in most teleost fishes [5,6,7,8,9,10,11]. Post-genome duplication independent gene loss has generated clustered paralog groups (PGs) that differ in gene number depending on the historical timing of gene losses relative to genome duplications [6,12,13,14,15].

In addition to variation in gene number within and among paralog groups, results from expression and functional genetic studies have shown that duplicate *Hox* genes can exhibit either similar or heterogenous expression patterns. In the cases in which the expression patterns among paralog group members differ, they often appear to be the result of sequence divergence within *cis*-regulatory elements [6,12,13,14,15,16,17,18,19,20,21]. A particular case of heterogeneous expression patterns exhibited by duplicate genes is that of *Hoxa2* and *b2* of tetrapods. Mouse *Hoxa2* is expressed in rhombomeres (r) 2–8 of the developing hindbrain and in the cranial neural crest cells (CNCCs) that delaminate from r4 and r6/7 and populate the second pharyngeal arch (PA2) and the posterior arches, respectively [22,23]. *Cis-*regulatory elements (CREs) that direct mouse *Hoxa2* expression in r2, r4 and r3, r5 and the CNCCs are located in exon 2 of *Hoxa2*, the intron and exon 1 of *Hoxa2*, and the *Hoxa3-a2* intergenic region, respectively [21,24,25,26,27,28,29,30,31,32,33,34]. There has been extensive evolutionary conservation of the regulatory circuitry that directs the hindbrain and PA patterning activity of *Hoxa2*, as similar gene regulation was observed for *Hox2* of lamprey (*Petromzon marinus*) [35]. By contrast, mouse *Hoxb2* is expressed in r3–8 of the hindbrain but not in the CNCCs [36]. The CREs that direct mouse *Hoxb2* in r3, r4 and r5 are located in the mouse *Hoxb3-b2* intergenic region [36,37,38]. The variation in mouse *Hoxa2* and *b2* regulation and expression are mirrored by their functional divergence. Knockout experiments have shown that *Hoxa2* controls the segmentation of the anterior hindbrain and the axonal guidance of the trigeminal (Vth) and facial (VIIth) cranial motor nerve axons out of r2 and r4, respectively, while *Hoxb2* controls the specification of the somatic motor component of the VIIth cranial nerve exiting r4 [39,40,41,42,43,44]. Further, *Hoxa2*, but not *Hoxb2*, is involved in patterning the craniofacial elements arising from PA2 and the posterior arches in tetrapods [22,23,45,46,47,48,49].

**Figure 1:**
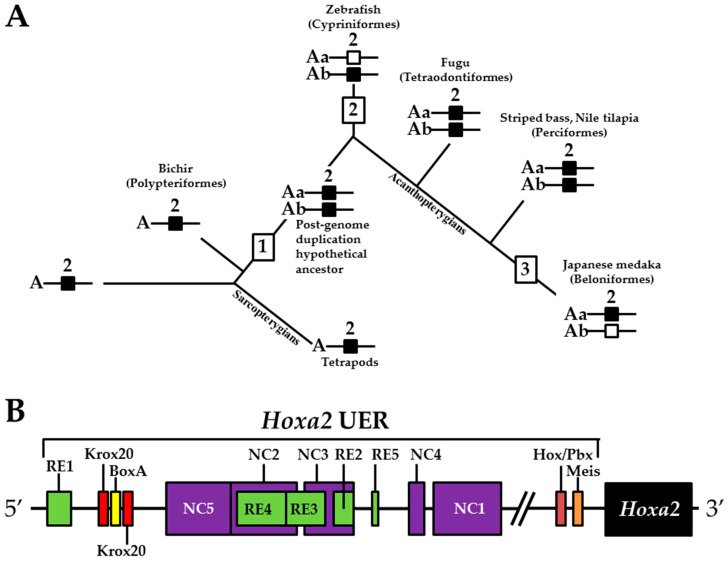
Evolution of *Hoxa2* gene complement and *Hoxa2* UER composition. (**A**) Phylogeny based on Steinke *et al.* (2006) [58]: (1) Genome duplication; (2) *hoxa2a* gene loss; and (3) *hoxa2b* gene loss. Filled in boxes represent functional *Hoxa2* genes. Open boxes represent loss of *Hoxa2* gene function. (**B**) Genomic map of the *Hoxa2* UER characterized in mouse. The genomic map was not drawn to scale. RE, rhombomeric element; NC, neural crest.

Phylogenetic reconstructions that include a whole genome duplication event at the incipient stage of teleost evolution, which occurred 350–220 million years ago [7,50,51,52,53,54], support a post-genome duplication ancestral *Hox* PG2 gene complement consisting of two *Hoxa2* genes, *hoxa2a* and *a2b* (Figure 1A). The absence of a *hoxa2a* gene in zebrafish, but not in any members of the superorder Acanthopterygii, suggests that the loss of *hoxa2a* was restricted to a clade including zebrafish but not the acanthopterygians [5]. Results based on cloning and expression analyses of several acanthopterygians, including striped bass (*Morone saxatilis*), Nile tilapia (*Oreochromis niloticus*), fugu (*Takifugu rubripes*) and Japanese medaka (*Oryzias latipes*), have shown that while striped bass, Nile tilapia and fugu have two functional *Hoxa2* genes, *hoxa2a* and *a2b*, medaka has just one, *hoxa2a* [12,14,19]. The paralog of medaka *hoxa2a* was shown to be a transcribed pseudogene (*ψhoxa2b*), wherein several premature stop codons were discovered in the region corresponding to exon 2 upstream of the putative homeodomain [12]. However, the amino terminal 138 amino acid sequence was shown to be intact and contained a conserved hexapeptide motif, indicative that transcripts arising from this sequence could be translated into a product with activities like hexapeptide motif-related Pbx binding [55]. Further, while the *hoxa2a* and *a2b* genes of most teleosts are expressed in a conserved manner in much of the hindbrain (r2–5), PA2 and the posterior arches similar to orthologous genes in tetrapods [6,12,14,19,28,30,45,49,56,57], the medaka *ψhoxa2b* was shown to be expressed in noncanonical *Hox* PG2 domains, including the ventral-most aspect of the neural tube, the distal mesenchyme of the pectoral fin buds and the caudal-most region of the embryonic trunk [12]. These noncanonical expression patterns may be a reflection of sequence changes resulting from relaxed selection within the medaka *ψhoxa2b* CREs following the mutational inactivation of the homeodomain but not the hexapeptide region of the medaka *hoxa2b* coding sequence [12].

The *Hoxa3*/*Hoxa2* intergenic region is the most extensively studied functional genomic region that directs *Hoxa2* gene expression. Results from comparative and functional genomic analyses in mouse and chick embryos have identified a highly conserved upstream enhancer region (UER) that is responsible for directing *Hoxa2* in r3 and r5 of the hindbrain and the CNCCs in the PA2 and the posterior arches (Figure 1B) [21,24,25,27,28,29,30,31,32]. Identified rhombomeric CREs within this region include binding sites for Krox20, a protein shown to be integral for hindbrain development [21,30,31,34,59]. Other rhombomeric elements, termed RE1-5 and BoxA, have been shown to function in conjunction with Krox20 in potentiating *Hoxa2* expression in r3 and r5 across diverse taxa [21,25,28,30,31,32], which is indicative that these elements may represent a regulatory kernel subcircuit, *sensu* Davidson and Erwin (2006) [60]. Five functional CREs that direct *Hoxa2* expression in the CNCCs populating the PA2 and posterior arches that are referred to as neural crest (NC) 1–5 have been identified in the mouse [27,29]. One of these elements, NC4, encodes a binding site for AP-2, a protein that is crucial for CNCC survival and chondrocyte differentiation within the PAs [27,61]. A recent analysis provides evidence for Hox/Pbx and Meis protein binding elements within the proximal promoter region of mouse *Hoxa2* [24]. The proteins that bind these elements have been shown to be crucial for craniofacial skeleton morphogenesis, [22,23,24,27,45,46,47,48,49,56,62,63,64,65,66].

The present study was undertaken to examine the activity of the medaka *hoxa2a* and *ψhoxa2b* UERs in their natural background, and to understand the relationship between sequence differences of the two medaka UERs relative to the striking differences in the expression of their cognate genes, one of which, *ψhoxa2b*, is expressed in development well outside the compartments in which expression is observed for any other osteichthyan *Hox* PG2 gene [12]. Most of the CREs mentioned above have recently been shown using comparative and functional genomic analyses to be present within the *hoxa3a-a2a* and *hoxa9b-a2b* intergenic regions of teleost fishes, except for the NC4 and NC1 elements [29]. At the time that the present study was conducted for the CREs upstream of medaka *hoxa2a* and *ψhoxa2b*, only a subset of *Hoxa2* UER-specific CREs were identified within the teleost *hoxa3a-a2a* and *hoxa9b-a2b* intergenic regions, and included, 5′ to 3′, Krox20, BoxA, RE4, RE3, RE2 and RE5 [21], hereafter referred to as the UER(K20-RE5). This sequence also included NC5, NC2, and NC3 sequences, which overlap many of the RE sequences mentioned above. Further, when the medaka *hoxa2a* UER(K20-RE5) was tested for its function in chick embryos through electroporation, no reporter gene expression was observed in the hindbrain or PAs [21]. However, the paralogous UER(K20-RE5) of medaka *ψhoxa2b* yielded reporter gene staining in r3 and r5 when it was electroporated into chick embryos [21]. These results were inconsistent with *in situ* hybridization results for medaka *hoxa2a*, which showed that this gene is expressed in r2–7 of the hindbrain, PA2 and the posterior PAs, and *ψhoxa2b*, which was shown to be expressed within noncanonical *Hox* PG2 expression domains, including the pectoral fin buds [12]. Therefore, consistent with the results of *in situ* hybridization, it was hypothesized that the medaka *hoxa2a* UER(K20-RE5) region would direct reporter gene expression in r3, r5, PA2 and the posterior PAs when expressed in medaka. Similarly, it was hypothesized that the paralogous region cloned from medaka *ψhoxa2b* would direct expression of the reporter gene in the noncanonical domains mentioned above. Contrary to expectations, reporter gene expression analyses showed that the medaka *hoxa2a* and *ψhoxa2b* UER(K20-RE5)s directed reporter gene expression in r4 and r3–7, respectively, as well as in the migratory CNCCs of the hyoid and post-otic streams and the post-migratory CNCCs in PA2 and the posterior arches. These results suggest that the functional nature of the UER(K20-RE5) has diverged between medaka *hoxa2a* and *ψhoxa2b*. Deletion mapping also revealed a short 88/89 base pair (bp) region within the medaka *hoxa2a* and *ψhoxa2b* UER(K20-RE5)s that includes consensus Hox/Pbx and Prep/Meis sites that are known to direct expression in r4 for mouse *Hoxb1*, *a2*, and *b2* and the CNCCs in mouse *Hoxa2* [21,24,26,27,33,37,67,68] and likely represents a deeply rooted core CRE element. This core CRE element is present in the regulatory sequences common to gnathostome *Hox* A clusters and its effects on expression of cognate *Hoxa2* genes appears to be influenced by interactions with cis-elements outside and sequence variation within the core element itself.

## 2. Materials and Methods

### 2.1. Tol2 Plasmid Construction

Transient and stable-line transgenic analyses employed the pT2AL200L150G plasmid vector for transmitting transposon insertions into medaka embryos (generous gift from Koichi Kawakami) [69]. This plasmid vector contains the *Xenopus laevis EFI-αS* promoter upstream of *enhanced green fluorescent protein* (*eGFP*), and was used for positive controls to determine if the *Tol2* transposon system functions similarly in medaka relative to other osteichthyans. Positive controls were performed by co-microinjection of pT2AL200R150G and transposon mRNA. Negative controls were performed by co-microinjection of constructs containing the *Xenopus laevis EFI-αS* promoter without transposon mRNA. Medaka contains roughly 20–30 copies of the *Tol2* element in its genome, and these controls were performed to determine whether constructs could be integrated into the medaka genome independently of exogenously translated *Tol2* mRNA [70]. The microinjection procedure used for medaka zygotes is described below.

In order to analyze the function of the CREs in control of the medaka *hoxa2a* and *ψhoxa2b* UER(K20-RE5)s in RNA expression, the full length *EFIα-S* promoter of the pT2AL200L150G was truncated to include only the region encompassing the TATA box so as to diminish the amount of transcription occurring from the *Xenopus* promoter and maximize transcription resulting from cloned sequences of putative *cis*-regulatory elements. The TATA box from the full length *EFIα-S* promoter was amplified from pT2AL200R150G using the forward primer 5′-GATAGGGATCCAGTTCTCAGGATCGGTCG-3′ (containing the underlined BamHI sequence) and the reverse primer 5′-GGGGGCTCGAGTATATAAAGGGTGGTTAAGG-3′ (containing the underlined XhoI sequence) using standard PCR procedures. To generate pTolTATA-βMCS the *EFIα-S* promoter and *β-globin* intron were removed from pT2AL200R150G using the flanking XhoI and BamHI sites, and the amplified TATA box containing sequence was then ligated into the pT2AL200R150G digested backbone using the compatible BamHI and XhoI termini. To facilitate the ligation of potential enhancer sequences upstream of the resulting *EFIα-S* TATA box, a polylinker containing restriction sites for SmaI, EcoRI, PmeI, and EcoRV was cloned into the 5′ XhoI site. The pTolTATA-βMCS vector was also co-microinjected with and without transposon mRNA to test the efficacy of this vector system in the medaka model.

### 2.2. Medaka Genomic DNA Extraction

Adult medaka were anesthetized with MS-222 (0.04 *w*/*v*) prior to genomic DNA extraction. Tissues from the trunk of the fish behind the anus were homogenized in 2 mL of DNA extraction buffer (0.5% SDS, 50 mM·Tris, 100 mM·NaCl, 20 mM disodium EDTA, pH 8.0) and the resulting homogenate was poured into sterile 15 mL Falcon tubes (Becton Dickinson Labware, Franklin Lakes, NJ, USA). The tissue homogenizer was washed with an additional 2.0 mL of DNA extraction solution, which was combined with the previous homogenate in the sterile 15 mL polypropylene tube. Five microliters of RNase A (20 mg/mL) (Sigma-Aldrich, St. Louis, MO, USA) was added to the cell homogenate and the tube was incubated for 30 min at 37 °C. The homogenate was then treated with 100 µL of protease K (10 mg/mL) (Life Technologies, Carlsbad, CA, USA) and incubated at 55 °C for 6 h. The RNase A and protease K-treated homogenate was transferred to 1.5 mL centrifuge tubes (Fisher Scientific, Pittsburgh, PA, USA) and centrifuged for 30 s at 14,000 rpm using an Eppendorf 5514 C centrifuge (Hauppauge, NY, USA). The resulting supernatant was then divided into two 500 µL aliquots in sterile 1.5 mL centrifuge tubes (Fisher Scientific), and DNA was purified by two Phenol Chloroform Isoamyl alcohol (PCI) extractions as follows: 500 µL of PCI (Life Technologies, Carlsbad, CA, USA) was added to each 1.5 mL tube and mixed until a homogeneous emulsion formed. Microcentrifuge tubes containing the emulsion were centrifuged for 30 s at 14,000 rpm, and the aqueous layer was removed from the organic layer and then placed in a sterile 1.5 mL microcentrifuge tube. After a second PCI extraction, the aqueous phase was treated with 5 M·NaCl, which was added to the aqueous extract, such that the final concentration of NaCl in the tube would be 0.3 M after the addition of 2 volumes of 100% ethanol (EtOH, Pharmco-Aaper, Brookfield, CT, USA). After addition of the ethanol, the tubes were mixed gently to allow the DNA to precipitate, after which the precipitated DNA was collected by centrifugation at 10,000× *g* for 1 min. The supernatant was removed by pipetting and the sedimented DNA was washed twice with 70% EtOH and incubated at 37 °C until dry. Once dry, the DNA was suspended in 100 µL of Nuclease Free Water (Life Technologies, Carlsbad, CA, USA). The quality of the genomic DNA was assayed by agarose gel electrophoresis on a 0.5% gel. The migration of the genomic DNA was compared to that of the DNA fragments in the High Molecular Weight standard (Life Technologies, Carlsbad, CA, USA).

### 2.3. Amplification of Medaka hoxa2a and ψhoxa2b UER(K20-RE5)s

Amplification of genomic DNA corresponding to *cis-*regulatory elements in the upstream DNA sequences, exons 1 and 2 and the intronic DNA of medaka *hoxa2a* and *ψhoxa2b* was performed using long-range PCR*.* Primers and their hybridization coordinates to medaka genomic DNA with respect to ATG start site of medaka *hoxa2a* and *ψhoxa2b* are listed in Table 1. All primers for all analyses were developed using published genomic sequences of the Japanese medaka [71]. The PCR products generated from long-range PCR were cloned into pCR II vectors (Life Technologies, Carlsbad, CA, USA) according to the manufacturer’s instructions. Confirmation and orientation of PCR products corresponding to inserts from plasmid genomic DNA clones were determined by restriction endonuclease digestion. These clones were then used to amplify the UER(K20-RE5)s of medaka *hoxa2a* and *ψhoxa2b*, which were tested for enhancer activity in the pTolTATA-βMCS plasmid vector.

### 2.4. PCR-Mediated Deletion Mutagenesis of the Medaka hoxa2a and ψhoxa2b UER(K20-RE5)s

DNA sequences that contained the UER(K20-RE5)s of medaka *hoxa2a* and *ψhoxa2b* were amplified using PCR primers listed in Table 1. These primers were used to generate a product referred to as the UER(K20-RE5), which encompassed sequence elements corresponding to Krox20, BoxA, RE4, RE3, RE2, RE5, NC2, NC3 and NC5, and was similar to the region tested in chicken embryos [21], and a series of nested deletions that were derived from the UER(K20-RE5). Specific primer pairs and their amplified genomic DNA products are listed in Table 2. All 5′-located primers started with the sequence 5′-GATCGATATC-3′ and 3′-located primers started with the sequence 5′-GATCGAATTC-3 to ensure that the PCR products could be digested with EcoRI and EcoRV restriction digestion enzymes and oriented 5′ to 3′ with respect to the TATA box and green fluorescent protein (GFP) sequences located downstream in the pTolTATA-βMCS vector. PCR products were purified using the QIAquick PCR purification kit (Qiagen, Valencia, CA, USA) according to the manufacturer’s instructions, restriction endonuclease digested with EcoRI and EcoRV and then ligated into the pTolTATA-βMCS plasmid vector using T4-DNA ligase (New England Biolabs, Ipswich, MA, USA). The pTolTATA-βMCS plasmid vector was pre-digested with EcoRI and EcoRV, purified using the QIAquick PCR sequence kit (Qiagen, Valencia, CA, USA) and treated with shrimp alkaline phosphatase (New England Biolabs, Ipswich, MA, USA) to remove phosphate groups at the digested sites of the vector to minimize vector religation. Plasmid vectors containing PCR-amplified genomic DNAs were transformed into *Escherichia coli (E. coli)* JM109 cells. *E. coli* cells were screened for recombinants by digestion of plasmid isolated from transformants with EcoRI and EcoRV after purification of plasmids using the GenElute HP Plasmid Miniprep kit (Sigma-Aldrich, St. Louis, MO, USA) according to the manufacturer’s instructions. Further confirmation of cloned PCR products in the pTolTATA-βMCS plasmid vector was performed using DNA sequencing of the inserts by dideoxyterminator sequencing chemistry (Big Dye v. 3.0, Applied Biosystems, Foster City, CA, USA) on a 3130xl Genetic Analyzer (Applied Biosystems, Foster City, CA, USA).

### 2.5. Microinjection of Medaka Embryos

Cultivation of medaka was performed as previously described [12]. Zygotes (stage 1) were collected from several females to ensure genetic variation per each injection experiment [72]. Zygotes were transferred to ice-cold 1× medaka embryo rearing medium (ERM) (17 mM·NaCl, 0.4 mM·KCl, 0.66 mM·MgSO_4_·7H_2_O, 0.27 mM·CaCl_2_·2H_2_O) between 30 min to 2 h to arrest development [73]. Zygotes were then physically transferred to specially fabricated agarose “corrals” that were pre-chilled at 4 °C and that hold the zygotes in an orientation appropriate for microinjection. The agarose corrals were 1 mm wide × 1 mm deep × 4 mm long and were made using 60 mL of 1.5% agarose (Sigma-Aldrich, St. Louis, MO, USA). To reduce high internal egg pressures when microinjecting, the agarose corrals were overlaid with 10 mL of 15% Ficoll 400 (Sigma-Aldrich, St. Louis, MO, USA) pre-chilled at 4 °C. Zygotes were placed in their corrals and were allowed to equilibrate for at least 40 min in 15% Ficoll 400 on ice prior to microinjection. Zygotes were microinjected using needles derived from filamented borosilicate glass capillaries that had an outer diameter of 1 mm and an inner diameter of 0.58 mm (World Precision Instruments, Sarasota, FL, USA). Glass needles were pulled on a Sutter P-97 needle puller (Novato, CA, USA) housing a 1.5 mm trough filament using the following parameters: heat of 250, pull of 200, velocity of 100, time of 200 and pressure of 400. To avoid clogging from chorions, the tips of the needles were beveled at a 45° angle using a Narishige Micro-grinder (Tokyo, Japan). After beveling the tips, glass needles were stored by embedding their mid-sections in rounded stripes of Play-Doh (Hasbro, Pawtucket, RI, USA) housed in 100 mm petri dishes with dampened Kimwipes (Kimberly Clark^®^, Irving, TX, USA) at 4 °C. The Play-Doh prevented glass needles from movement and breakage in the Petri plates. The dampened Kimwipe maintained a moist environment inside the Petri dishes and kept the stored needles from clogging at the beveled ends. Microinjection of solutions into medaka zygotes with beveled needles was performed using a PV 820 pneumatic picopump (World Precision Instruments, Sarasota, FL, USA). Twenty pounds per square inch (psi) of eject pressure and 3 psi of hold pressure were used for the medaka zygote microinjection process. Zygotes were microinjected with the following solutions for analyzing transient transgenic embryos: 25 ng/µL plasmid DNA, 25 ng/µL *Tol2* transposase RNA, 1× Yamamoto Buffer (128 mM·NaCl, 2.7 mM·KCl, 1.8 mM·CaCl_2_, 0.24 mM·NaHCO_3_, pH 7.3). After microinjection, embryos were carefully removed from their individual agarose corrals and transferred in approximately 1 mL of 15% Ficoll to a Petri dish containing 20 mL 1× ERM. After transfer they were allowed to equilibrate to less than 1% Ficoll for roughly 2 h at room temperature (RT) without shaking. The medium housing the embryos was then replaced with fresh 1× ERM and the embryos were incubated at 28.5 °C to continue development.

### 2.6. Generation and Visualization of Transient and Stable-Line Transgenic Medaka Embryos

Microinjected embryos were reared in 1× ERM at 28.5 °C. After 24 h, embryos were visualized under a Leica dissecting microscope. Dead eggs and embryos that showed extremely defective morphologies (*i.e*., gastrulation defects) were discarded. All other embryos were transferred in 1× ERM containing 0.1 mM phenylthiourea (Sigma-Aldrich, St. Louis, MO, USA) to reduce pigmentation [74]. Embryos were visualized for *eGFP* expression using a Zeiss inverted compound microscope (Thornwood, NY, USA) at selected times during development, focusing primarily on developmental stage 29/30 (74–82 hpf [72]). At stage 29/30, the hindbrain and pharyngeal arches are easily distinguished morphologically in medaka embryos when they are in the chorion. Since embryos at stage 29/30 have guanophores that auto-fluoresce under GFP filters, illumination of specimens using both GFP and rhodamine filters was performed to differentiate positive *eGFP* signal from auto-fluorescing pigmentation. Images of *eGFP* expression were processed using Axiovision AC 4.4 software. Depending on the strength of the *eGFP* signal, camera exposure times ranged from 1 to 2 s for transient transgenic embryos. Brightfield images were also taken of medaka embryos to determine the relative origin of the *eGFP* signal within the hindbrain and pharyngeal arches in transient transgenic embryos. The developing otic vesicle was used as a morphological landmark to determine whether the *eGFP* signal was occurring anteriorly or posteriorly in the hindbrain. Rhombomeres 3 and 4 develop dorsal to the anterior region of the otic vesicle while r5, r6 and r7 develop above the mid- to posterior region of the otic vesicle.

Transient transgenic medaka embryos that were observed to be positive for *eGFP* expression in the hindbrain and pharyngeal arches showed mosaic expression in comparisons among embryos. For this reason, transient transgenic embryos that showed strong *eGFP* signal in the hindbrain and/or pharyngeal arches were reared to adulthood and mated with wild-type fish. We observed that more robust *eGFP* expression patterns were detected in the hindbrain and pharyngeal arches of stable-line transgenic medaka embryos compared to transient transgenics. Transient transgenic embryos were reared until hatching in 1× ERM at 28.5 °C. Hatched embryos were reared in 1× ERM in breeding nets in 5 gallon tanks until they were large and strong enough to swim against filter-generated currents. F1 medaka embryos were assayed for *eGFP* expression in the hindbrain and pharyngeal arches according to the procedure outlined above for transient transgenic embryos.

### 2.7. Whole-Mount in Situ Hybridization

To corroborate the results of *eGFP* expression using a system independent of fluorescence, the expression of *eGFP* in embryos was visualized using *in situ* hybridization analysis with an anti-*eGFP* riboprobe. This method allowed for the visualization of *eGFP* expression in the absence of fluorescence originating from auto-fluorescing pigment cells, which was often the case when embryos were visualized under a GFP filter. We used whole-mount *in situ* hybridization to visualize *eGFP* transcripts at developmental stages 22 (nine somites), 29/30 and 34 (121 hpf) [72]. We assayed embryos at stage 22 because we wanted to determine if the CREs of the medaka *hoxa2a* and *ψhoxa2b* UER(K20-RE5)s were directing reporter gene expression in the migratory CNCCs of the hyoid and post-otic streams, at stage 29/30 to visualize the exact rhombomeres and pharyngeal arches that were expressing *eGFP* and at stage 34 to determine if the CREs of the medaka *hoxa2a* and *ψhoxa2b* UER(K20-RE5)s were directing expression in post-migratory CNCCs at the chondrogenic stages of pharyngeal arch development.

Embryos from stable-line transgenic medaka fish were collected, reared, anesthetized, fixed in 4% paraformaldehyde (PFA) and dehydrated as previously described [12]. Medaka embryos were developmentally staged according to Iwamatsu (2004) [72]. Whole-mount *in situ* hybridization was performed according to Davis *et al.* (2008) [12]. All experiments used digoxigenin (DIG)-labeled sense and antisense riboprobes that were produced and purified according to Scemama *et al.* (2006) [19]. Sense riboprobes were used in control experiments to assess nonspecific binding. Development of DIG-labeled probe signal, examination of embryos and digital photography of embryos was performed as described in Scemama *et al.* (2006) [19]. Morphological landmarks, including the midbrain/hindbrain boundary, rhombomeres (r), otic vesicles (OV), pectoral fins (PF), pharyngeal arches (PA) and somites (s) within developing embryos were used to define the location of *eGFP* expression. *eGFP* signal was also determined using double whole-mount *in situ* hybridization assays with DIG-labeled antisense-*eGFP* riboprobes and DIG-labeled antisense-*hoxd3a* riboprobes or fluorescein-labeled *hoxb1a* riboprobes. Medaka *hoxd3a* is expressed in r6–8 of the hindbrain and *hoxb1a* is expressed in r4 [13,16]. Production of fluorescein riboprobes and double whole-mount *in situ* hybridization using DIG and fluorescein-labeled riboprobes was performed as documented in Scemama *et al.* (2006) [19].

### 2.8. Comparative Genomic Sequence Analysis

Genomic DNA sequences corresponding to the UER(K20-RE5)s of medaka *hoxa2a* and *ψhoxa2b* that were shown to be required for directing reporter gene expression in the rhombomeric and PA embryonic domains using functional genomic analyses outlined above were examined for putative transcription factor binding sites using JASPAR [75]. We also performed a comparative genomic sequence analysis of the UER(K20-RE5)s between medaka *hoxa2a* and *ψhoxa2b* and the UER(K20-RE5)s of other vertebrate *Hoxa2* genes using the software programs Dialign-TX and CLUSTALX in order to determine if the putative transcription factor binding sites were conserved in other vertebrates [76,77]. The *Hoxa2* UER(K20-RE5)s from genomic sequences of medaka (accession numbers AB232918 and AB232919), fugu (accession numbers DQ481663 and DQ481664), zebrafish (accession number AL645795, direct submission), Nile tilapia (AF533976 and GCA_000188235.1), bichir, mouse (accession number: NC000072) , human (accession number: NG012078, direct submission), chicken (accession number: AC163712, direct submission), ceolacanth (accession number: FJ497005), horn shark (accession number: AF224262), dogfish (accession number: FQ032658), and western clawed frog (accession number: NW004668239) were used for sequence comparison [71,78,79,80,81,82,83,84,85]. All default and recommended parameters were used in the Dialign-TX and CLUSTALX programs for conducting genomic DNA sequence comparisons.

## 3. Results

### 3.1. Validation of the Tol2 Transposon System for Medaka Embryos

**Figure 2:**
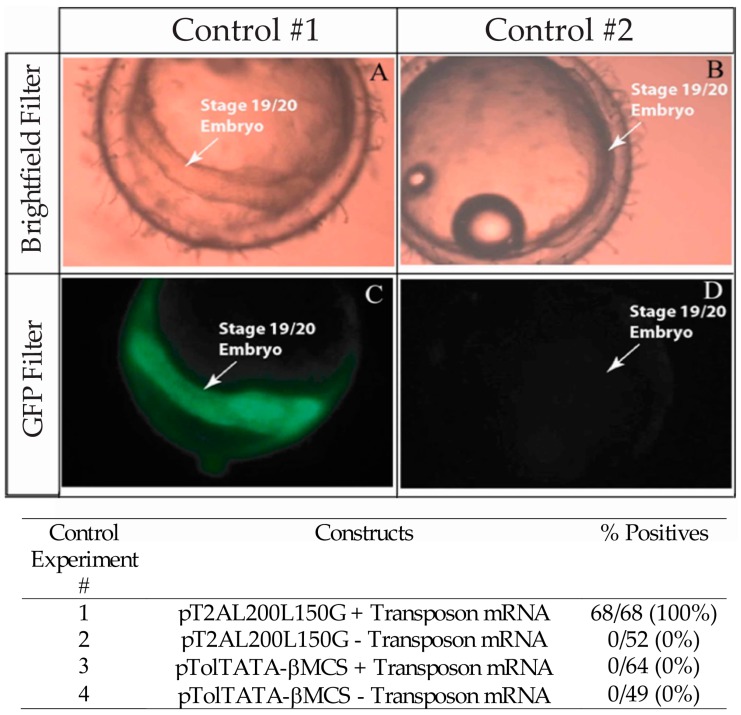
Control experiments used for the validation of the *Tol2* transposon system for medaka embryos. (**A**) Brightfield image of representative positive control embryo (pT2AL200L150G + Transposon mRNA); (**C**) Green fluorescent protein (GFP) filter image of the same embryo shown in A; (**B**) brightfield image of representative negative control embryo (pT2AL200L150G − Transposon mRNA); and (**D**) GFP filter image of the same embryo shown in B. No embryos in any of the other negative control experiments showed eGFP expression (pTolTATA-βMCS + Transposon mRNA or pTolTATA-βMCS-Transposon mRNA).

In order to determine if the *Tol2* transposon system functions similarly in medaka compared to zebrafish, we performed experiments using the pT2AL200L150G vector. This plasmid vector contains the *Xenopus laevis* eFI-*α*S promoter upstream of *eGFP*. We expected that positive control embryos, which were co-microinjected with pT2AL200L150G vector and *Tol2* transposon mRNA, would show green fluorescence during development. However, we were unsure if negative control embryos, which were microinjected solely with pT2AL200L150G vector, would not exhibit green fluorescence, since the *Tol2* element is present within the medaka genome [70]. All positive control embryos showed strong reporter gene expression throughout the body of the medaka embryos (68/68; 100%) (Figure 2A,C) while negative control embryos failed to show any reporter gene expression (0/52; 0%) (Figure 2B,D), which suggested that *eGFP* expression was not generated from microinjected vectors in the absence of exogenous *Tol2* transposon mRNA. Further, medaka zygotes microinjected with the pTolTATA-βMCS vector alone (0/49, 0%) or co-microinjected with vector and transposon mRNA (0/64, 0%) lacked detectable fluorescence (0/64, 0%) (Figure 2B,D). These results showed that the *Tol2* transposon system and the pTolTATA-βMCS vector can be used in medaka embryos for studying *cis-*regulatory element control of gene expression.

### 3.2. Functional Genomic Analysis of the Medaka Hoxa2a UER(K20-RE5)

Medaka *hoxa2a* is expressed in a relatively conserved manner in the hindbrain and pharyngeal arches similar to *hoxa2a* and *a2b* genes from teleosts, including zebrafish, tilapia, striped bass and fugu and *Hoxa2* genes of tetrapods, including the mouse, chicken and frog [6,12,14,19,23,24,31,45,49,56,57]. Based on this extensively conserved pattern of expression, we hypothesized that the medaka *hoxa2a* UER(K20-RE5) would direct reporter gene expression in r3 and r5 of the hindbrain and in the CNCCs entering PA2 and the posterior arches.

**Figure 3:**
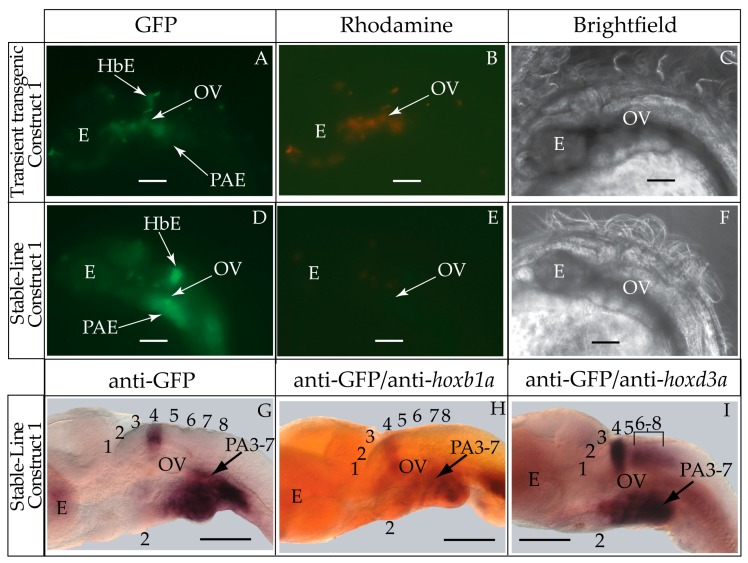
Transient (**A**–**C**) and stable-line (**D**–**I**) transgenic data from the medaka *hoxa2a* UER(K20-RE5) (Construct #1). (**A**–**F**) Images of transient (**A**–**C**) and stable-line (**D**–**F**) transgenic embryos at stage 29/30 (72–84 hpf) were taken using: GFP (**A**,**D**); rhodamine (**B**,**E**); and brightfield (**C**,**F**) filters. All embryos are still in their chorions and are positioned with their anterior sides to the left and their lateral sides to the reader. (**G**–**I**) Whole-mount *in situ* hybridization of medaka embryos using DIG-labeled anti-*eGFP* riboprobe (**G**). DIG-labeled anti-*eGFP* riboprobe with fluorescein-labeled anti-*hoxb1a* riboprobe (**H**) and DIG-labeled anti-*eGFP* and anti-*hoxd3a* riboprobes (**I**). Embryos were mounted with anterior sides facing left and lateral sides facing the reader. Rhombomere numbers are indicated by black numbers above the dorsal sides of the embryos. Pharyngeal arch 2 is indicated by the number 2 below the ventral sides of the embryos. Medaka *hoxd3a* expressing rhombomeres (r6–8) are indicated by the bracket in I. E, eye; HbE, hindbrain expression; OV, otic vesicle; PA, pharyngeal arch; PAE, pharyngeal arch expression. Scale bars equal 0.1 mm.

**Figure 4:**
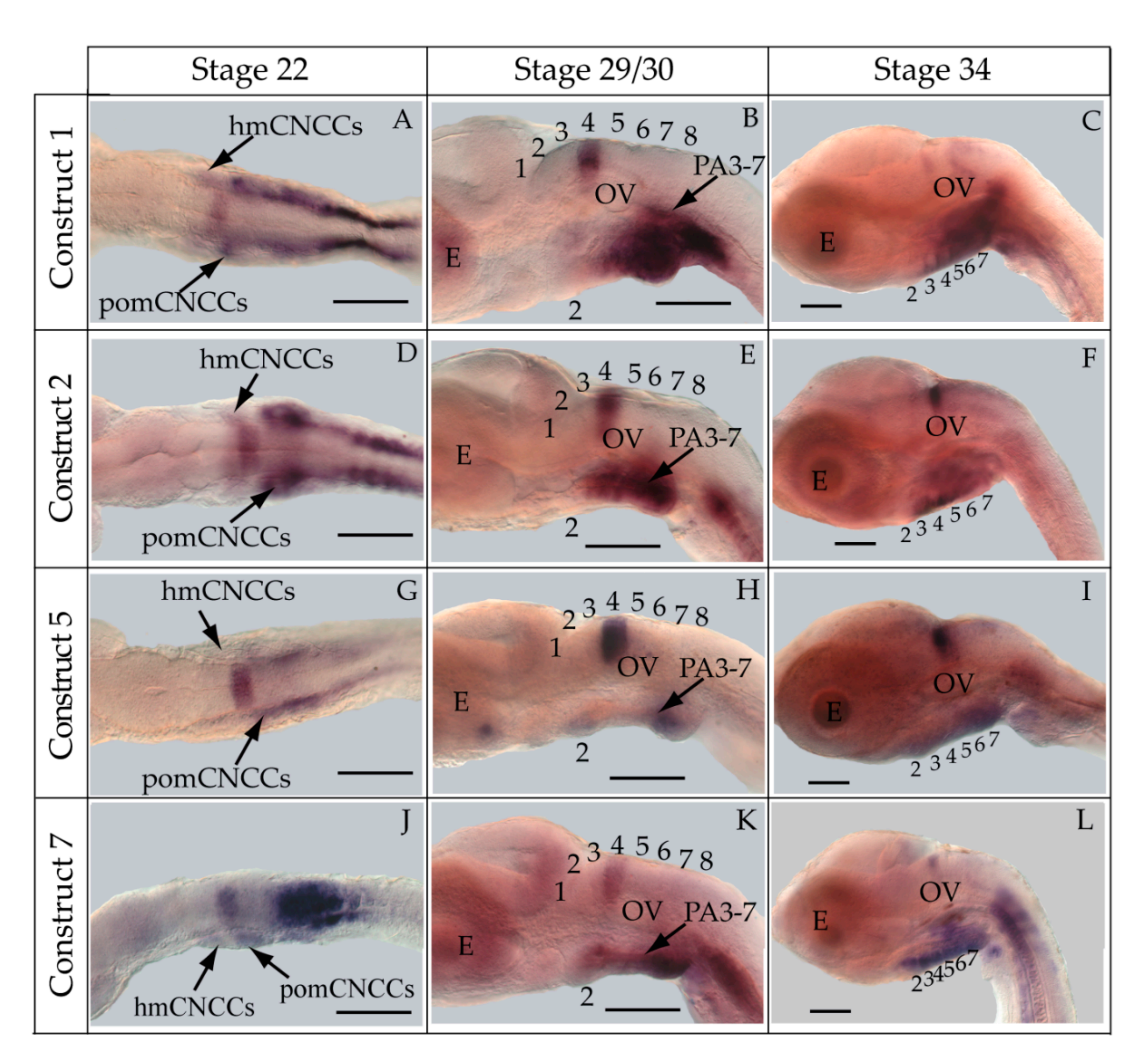
Whole-mount *in situ* hybridization of *eGFP* in stable-line *hoxa2a* UER(K20-RE5) transgenic medaka embryos generated with Construct #1 (**A**–**C**); Construct #2 (**D**–**F**); Construct #5 (**G**–**I**); and Construct #7 (**J**–**L**) at stages 22 (9 s) (**A**,**D**,**G**,**J**); 29/30 (72–84 hpf) (**B**,**E**,**H**,**K**); and 34 (121 hpf) (**C**,**F**,**I**,**L**). (**A**,**D**,**G**,**J**) Embryos were mounted with their anterior sides facing left and their dorsal sides facing the reader. (**B**,**C**,**E**,**F**,**H**,**I**,**K**,**L**) Embryos were mounted with their anterior sides facing left and lateral sides facing the reader. (**B**,**E**,**H**,**K**) Images are magnified to show rhombomere placement. Rhombomere numbers are indicated by black numbers above the dorsal sides of the embryos. Pharyngeal arches are indicated by black numbers below the ventral sides of the embryos. E, eye; hmCNCCs, hyoid migratory cranial neural crest cells; OV, otic vesicle; PA, pharyngeal arch; pomCNCCs, post-otic migratory cranial neural crest cells. Scale bars equal 0.1 mm.

Contrary to our hypothesis, transient and stable-line transgenic medaka embryos showed that the construct containing the medaka *hoxa2a* UER(K20-RE5) (Construct #1) directed reporter gene expression in r4 of the hindbrain and in PA2 and the posterior PAs (Figure 3A–G). Even though we observed *eGFP* expression in the hindbrain (87.5%) and pharyngeal arches (87.5%) in a high percentage of transgenic embryos (Table 2), both direct fluorescence and whole-mount *in situ* hybridization with riboprobes directed against eGFP expressed in stable-line transgenic embryos showed that the *eGFP* expression in the hindbrain was restricted to r4 (Figure 3D–G). This was unexpected given that the UERs of chicken and mouse *Hoxa2* or zebrafish *hoxa2b* directed reporter gene expression in r3 and r5 [21,25,27,28,29,30,31,32]. This restricted and unusual pattern of expression prompted us to examine the validity of rhombomere assignments. To authenticate the rhombomere assignments, additional whole-mount *in situ* hybridization experiments were conducted using medaka *hoxb1a* and *hoxd3a*, which are expressed in r4, and r6–8 of the hindbrain, respectively, but not in the pharyngeal arches. [13,16]. Medaka *hoxb1a* antisense riboprobes were labeled with fluorescein. Both fluorescein-labeled *hoxb1a* and DIG-labeled *eGFP* riboprobes were observed to hybridize to their mRNA targets in r4 and to overlap the region that hybridizes to the *hoxa2a* UER(K20-RE5) reporter construct #1. (Figure 3H). When DIG-labeled *eGFP* and *hoxd3a* antisense riboprobes were used in tandem for *in situ* hybridization experiments, a one-rhombomere gap without any DIG-stained cells between the *eGFP* and *hoxd3a* expressing rhombomeres was observed (Figure 3I). These results are consistent with expression of *eGFP* in r4 with a gap in expression corresponding to r5 and *hoxd3a* expression in r6–8 (Figure 3I), and show that the medaka *hoxa2a* UER(K20-RE5) reporter expression was restricted to just r4. Further, *eGFP* riboprobes were observed to hybridize to their mRNA targets in the migratory CNCCs of the hyoid and post-otic streams at developmental stage 22, the post-migratory CNCCs in PA2 and the posterior pharyngeal arches at stage 29/30 and the chondrogenic CNCCs in PA2 and the posterior arches at stage 34 (Figure 4A–C). These results confirm that the medaka *hoxa2a* UER(K20-RE5) construct #1 directs reporter gene expression in the CNCCs. Moreover, the presence of reporter gene expression within the CNCCs in the PAs of medaka is consistent with results from previous studies for orthologous UER sequences from mouse *Hoxa2*, fugu *hoxa2a* and zebrafish *hoxa2b* [24,27,29]. Interestingly, we observed more robust staining in *in situ* hybridization analyses using *eGFP* riboprobes in the posterior PAs than in PA2, which suggests that the sequence elements in the medaka *hoxa2a* UER(K20-RE5) construct #1 are expressed more strongly in the posterior PAs than for PA2, which is the converse of that observed for the cognate gene transcripts. 

To pinpoint the CREs responsible for directing the r4 and PA-specific reporter gene expression in medaka embryos, a comparison of expression in transient and stable-line transgenic embryos generated with a series of nested deletion constructs extending from the 5′- and 3′-ends of the medaka *hoxa2a* UER(K20-RE5) was performed (Constructs #2-7) (Table 2). These deletions eliminated regions corresponding to previously mapped enhancer elements including Krox20, BoxA, RE4, RE3, RE2 and RE5 [21,25,28,29,30,31,32]. Similar to transient transgenic embryos generated with Construct #1, microinjection of constructs that included 5′-deletions of Krox20 and BoxA binding sites (Construct #2) or Krox20, BoxA and RE4 (Construct #3) yielded high percentages of transient transgenic embryos with *eGFP* expression in the hindbrain (76%, Construct #2; 80%, Construct #3) and PAs (67%, Construct #2; 84%, Construct #3) (Table 2). Further, stable-line transgenic embryos generated with Construct #2 showed similar *eGFP* expression patterns to transgenic embryos generated with constructs containing the entire medaka *hoxa2a* UER(K20-RE5) (Construct #1). Specifically, we observed *eGFP* expression in r4 of the hindbrain, the migratory CNCCs of the hyoid and post-otic streams and the post-migratory and chondrogenic CNCCs of PA2 and the posterior PAs (Figure 4D–F). Like the full-length UER(K20-RE5) (construct #1) we observed stronger *eGFP* expression in the posterior PAs than in PA2. Unfortunately, we did not have success in obtaining stable-line transgenic embryos using Construct #3 (data not shown). Further, the extreme mosaic nature of the expression of the transient transgenic embryos treated with Construct #3 precluded us from drawing conclusions about the expression patterns. A much lower percentage of transient transgenic embryos generated with Construct #4, which included deletions of Krox20, BoxA, RE4 and RE3 sequences and only retained RE2 and RE5 sequences (Table 2), showed *eGFP* expression in the hindbrain (15%) and PAs (15%) when compared to embryos generated with Constructs #1, 2 and 3. Further, these embryos showed *eGFP* levels of expression that were barely detectable in the hindbrain and PAs and did not allow us to draw any conclusions of the expression patterns (data not shown). Unfortunately, we did not obtain any stable-line transgenic embryos treated with Construct #4 (data not shown). These results suggested that sequence elements downstream of RE4 within the medaka *hoxa2a* UER(K20-RE5), specifically, RE3, RE2 and RE5, are necessary for directing medaka *hoxa2a* expression in r4 and the CNCCs of PA2 and the posterior arches.

The microinjection of constructs with the 3′-specific deletion of RE5 from the medaka *hoxa2a* UER(K20-RE5) but retention of Krox20, BoxA, RE4, RE3 and RE2 (Construct #5) yielded similar results to that of Construct #1, namely a high percentage of transient transgenic embryos with *eGFP* expression in the hindbrain (84%) and PAs (84%) (Table 2). Further, stable-line transgenic embryos generated with Construct #5 showed *eGFP* expression in r4 of the hindbrain, the migratory CNCCs of the hyoid and post-otic streams, and the post-migratory and chondrogenic CNCCs in PA2 and posterior arches (Figure 4G–I). However, embryos injected with Construct #5 showed reduced *eGFP* expression levels in the posterior PAs when compared to embryos treated with Constructs #1 and #2 (Figure 4B,E,H). Interestingly, embryos injected with Construct #6, in which both RE2 and RE5 were deleted but Krox20, BoxA, RE4, and RE3 were retained, did not show any *eGFP* expression in the hindbrain (0%) or in the pharyngeal arches (0%) (Table 2). Overall, these results showed that the sequence containing the elements corresponding to RE3 and RE2 of the medaka *hoxa2a* UER(K20-RE5) is required for r4 expression but the surrounding elements corresponding to Krox20, BoxA, RE4 and RE5 are not. Similar results were also observed for the reporter gene expression in the PAs, such that the loss of either RE3 or RE2 sequences yielded a loss in CNCC expression (Constructs #4 and 6) in transient transgenic embryos.

The aforementioned *eGFP* expression patterns obtained from the nested deletion constructs of the medaka *hoxa2a* UER(K20-RE5) prompted us to develop a construct that included the RE3 and RE2 elements only (Construct #7; Table 2). This construct was 89 bp in length and spanned from genomic bp positions −1392 to −1303 (Table 2). As expected based on the results from earlier tested constructs containing both RE3 and RE2 (Constructs #1, 2, 3 and 5), a high percentage of transient transgenic medaka embryos generated with Construct #7 showed *eGFP* expression in the hindbrain (84%) and PAs (84%) (Table 2). Stable-line transgenic medaka embryos confirmed that the sequence elements within this region directed *eGFP* expression in r4 of the hindbrain, the migratory CNCCs of the hyoid and post-otic streams and the post-migratory and chondrogenic CNCCs of PA2 and the posterior arches (Figure 4J–L). Further, in comparison to stable-line transgenic embryos generated with constructs containing RE3, RE2 and other surrounding elements (Constructs #1, 2 and 5), embryos generated with Construct #7 showed much more robust *eGFP* expression in PA2. Altogether, these results indicate that this 89 bp sequence contains elements necessary and sufficient to direct *Hoxa2* expression in r4, PA2 and the posterior PAs in the absence of other elements.

**Figure 5:**
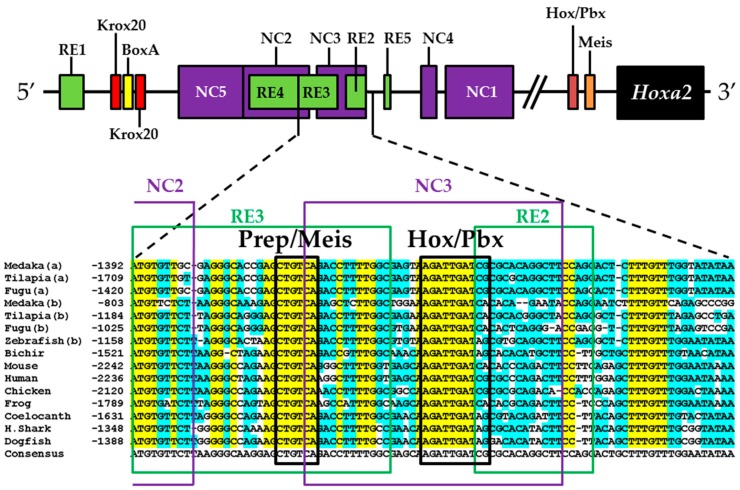
Comparative genomic sequence analysis of the 89 bp DNA fragment of the medaka *hoxa2a* UER(K20-RE5) (top sequence) that directs expression in r4 and the CNCCs. This sequence was compared to orthologous DNA sequences upstream of *hoxa2a* (denoted by a in parentheses after species name) and *hoxa2b* (denoted by b in parentheses after species name) of teleosts, *Hoxa2* of tetrapods and *Hoxa2* of coelacanth, horn shark and dogfish. Numbers correspond to genomic base pair positions relative to the ATG start site of the *Hoxa2* genes. The schematic diagram above the sequences corresponds to the mouse *Hoxa2* UER and the relative location of the 89-bp DNA fragment of the medaka *hoxa2a* r4/CNCC-specifying element. The schematic is not drawn to scale. Base pairs colored in yellow correspond to complete conservation at particular sites across all sequences examined. Base pairs colored in blue represent the majority of the sequences containing specific base pairs at specific sites. Purple boxed regions and green boxed elements correspond to neural crest and rhombomeric elements defined in the mouse *Hoxa2* UER [27,28]. Black boxed regions correspond to transcription factor binding sites identified in this study. A consensus sequence was derived from the aligned sequences. NC, neural crest; RE, rhombomeric element.

To identify putative transcription factor binding sites in the 89 bp region of the medaka *hoxa2a* UER(K20-RE5), hereafter referred to as the r4/CNCC-specifying element or core element, we performed a comparative genomic sequence analysis of this DNA sequence fragment with orthologous sequences from *Hoxa2* UERs of divergent gnathostomes , including horn shark, dogfish, coelacanth, frog, chicken, mouse, human, bichir, zebrafish, tilapia, fugu and medaka. Sequence alignments revealed several regions that were highly conserved among gnathostome *Hoxa2* UER genomic sequences but restricted to the region corresponding to the r4/CNCC-specifying element from medaka (Figure 5). Analysis of these sequences showed that they corresponded to a Prep/Meis binding site (5′-CTGTCA-3′) beginning at bp position −1371 of the medaka *hoxa2a* UER(K20-RE5) and a Hox/Pbx binding site (5′-AGATTGATCG-3′) beginning at bp postion −1349 (Figure 5). Interestingly, the Prep/Meis and Hox/Pbx sites of the medaka *hoxa2a* UER(K20-RE5) were observed to be identical in sequence to the functionally mapped Prep/Meis and Hox/Pbx binding sites in the mouse *Hoxb3-b2* intergenic region [37], which were shown to direct mouse *Hoxb2* expression in r4 and the migratory CNCCs [37]. To further support the concept of the r4/CNCC element as a core element, it is important to note that Hox/Pbx and Meis binding sites located upstream of mouse *Hoxa2*, but downstream of the sequence orthologous to the medaka r4/CNCC element, were shown to direct *Hoxa2* expression in the CNCCs of PA2 and the posterior arches [24]. Likewise, Hox/Pbx and Prep/Meis binding sites located in Exon 1 and the intron of *Hoxa2* of chicken and mouse were shown to direct expression in r4 [21,26,33]. Therefore, it is possible that flanking sequences not analyzed in this study that are orthologous to sequences of the *Hoxa2* UERs in other gnathostomes function to redirect the intrinsic r4 activity of the r4/CNCC-specifying element to r3 and r5.

### 3.3. Functional Genomic Analysis of the Medaka ψHoxa2b UER(K20-RE5)

Unlike the conserved hindbrain and pharyngeal arch expression patterns common to many teleost *hoxa2a* and *a2b* and tetrapod *Hoxa2* genes, medaka *ψhoxa2b* is expressed in noncanonical *Hox* PG2 domains, which include the caudal-most region of the embryonic trunk, the ventral-most aspect of the neural tube and the distal regions of the pectoral fin buds [12]. Based on the divergence in expression observed in comparisons of medaka with other teleost and tetrapod *Hoxa2* genes, we hypothesized that the UER(K20-RE5) of medaka *ψhoxa2b* would direct expression in the noncanonical *Hox* PG2 domains instead of the hindbrain or CNCCs.

**Figure 6:**
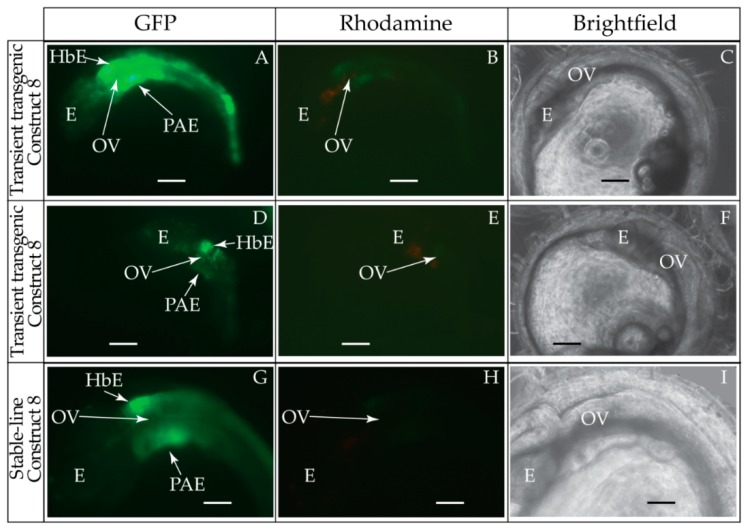
Transient (**A**–**F**) and stable-line (**G**–**I**) transgenic data from the medaka *ψhoxa2b* UER(K20-RE5)-(Construct #8). (**A**–**I**) Images of transient transgenic embryos at stage 29/30 (72–84 hpf) were taken using: GFP (**A**,**D**,**G**); rhodamine (**B**,**E**,**H**); and brightfield (**C**,**F**,**I**) filters. Transient transgenic analyses show varying degrees of *eGFP* signal between embryos (compare **A** and **D**). All embryos are still in their chorions and are positioned with their anterior sides to the left and their lateral sides to the reader. E, eye; HbE, hindbrain expression; OV, otic vesicle; PAE, pharyngeal arch expression. Scale bars equal 0.1 mm.

**Figure 7:**
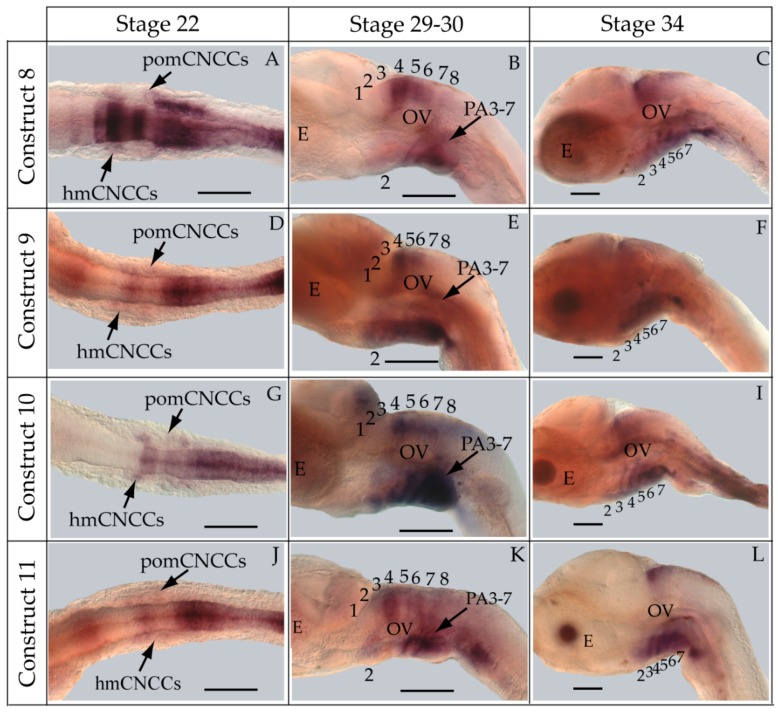
Whole-mount *in situ* hybridization of *eGFP* in stable-line *ψhoxa2b* UER(K20-RE5) transgenic medaka embryos generated with: Construct #8 (**A**–**C**); Construct #9 (**D**–**F**); Construct #10 (**G**, **H** and **I**); and construct #12 (**J**–**L**) at stages 22 (9 s) (**A**,**D**,**G**,**J**); 29/30 (72–84 hpf); (**B**,**E**,**H**,**K**); and 34 (121 hpf) (**C**,**F**,**I**,**L**). (**A**,**D**,**G**,**J**) Embryos were mounted with their anterior sides facing left and their dorsal sides facing the reader. (**B**,**C**,**E**,**F**,**H**,**I**,**K**,**L**) Embryos were mounted with their anterior sides facing left and lateral sides facing the reader. (**B**,**E**,**H**,**K**) Images are magnified to show rhombomere placement. Rhombomere numbers are indicated by black numbers above the dorsal sides of the embryos. Pharyngeal arches are indicated by black numbers below the ventral sides of the embryos. E, eye; hmCNCCs, hyoid migratory cranial neural crest cells; OV, otic vesicle; PA, pharyngeal arch; pomCNCCs, post-otic migratory cranial neural crest cells. Scale bars equal 0.1 mm.

Contrary to our hypothesis, transient and stable-line transgenic medaka embryos showed that the 397 bp construct containing the entire medaka *ψhoxa2b* UER(K20-RE5) (Construct #8, Table 2) directed reporter gene expression in the hindbrain and PAs (Figure 6A–I, Table 2) instead of the expected noncanonical *Hox* PG2 expression domains, such as the pectoral fins. We observed a high percentage of transient transgenic embryos showing *eGFP* expression in the hindbrain (90%) and pharyngeal arches (90%) (Table 2). Whole-mount *in situ* hybridization analyses of stable-line transgenic embryos generated with construct #8 showed that *eGFP* was expressed in r3–7 of the hindbrain, the migratory CNCCs of the hyoid and post-otic streams, the post-migratory CNCCs in PA2 and the posterior arches and the chondrogenic CNCCs in PA2 and the posterior arches (Figure 7A–C). Interestingly, in transgenic reporter gene assays conducted in chicken embryos, a very similar construct containing a region corresponding to the the medaka *ψhoxa2b* UER(K20-RE5) used in this study, and also lacking the RE1 sequence element, directed reporter gene expression in r3 and r5 but not in the CNCCs, the pharyngeal arches or the noncanonical expression domains observed for *ψhoxa2b* expression in medaka [12,21].

To precisely map the functional activity of the medaka *ψhoxa2b* UER(K20-RE5) on control of expression, a series of nested deletion constructs from the medaka *ψhoxa2b* UER(K20-RE5) that selectively eliminated enhancer elements and transcription factor binding sites similar to those that were deleted in the *hoxa2a* UER(K20-RE5) series of nested deletions was performed (Constructs #9–13) (see Table 2). Similar to embryos microinjected with Construct #8, microinjection of constructs that included 5′-deletions of Krox20 and BoxA binding sites (Construct #9) or Krox20, BoxA and RE4 (Construct #10) yielded high percentages of transient transgenic embryos with *eGFP* expression in the hindbrain (90%, Construct #9; 97%, Construct #10) and PAs (90%, Construct #9; 87%, Construct #10) (Table 2). Stable-line transgenic embryos generated with Constructs #9 and #10 showed similar *eGFP* expression pattern to those treated with Construct #8, wherein reporter gene expression was observed in r3–7 of the hindbrain, the migratory CNCCs of the hyoid and post-otic streams, the post-migratory CNCCs in PA2 and the posterior arches and the chondrogenic CNCCs of PA2 and the posterior arches (Figure 7D–I). Similar to the medaka *hoxa2a* UER(K20-RE5) analysis with Construct #4 in which the Krox20, BoxA, RE4 and RE3 sequences were eliminated, a lower percentage of transient transgenic medaka embryos generated with Construct #11, which included the same deletions, showed *eGFP* expression in the hindbrain (52%) and pharyngeal arches (52%) when compared to embryos generated with Constructs #8, 9 or 10 (Table 2). Further, *eGFP* expression was almost undetectable in embryos generated with Construct #11 (Data not shown). Unfortunately, no stable-line transgenic embryos generated with Construct #11 showed any detectable *eGFP* expression in the hindbrain or pharyngeal arches (Data not shown). These results, like those for the medaka *hoxa2a* UER(K20-RE5), suggest that sequence elements downstream of the RE4 element function to direct medaka *ψhoxa2b* in r3–7, PA2 and the posterior arches. The microinjection of constructs with the 3′-specific deletion of RE5 from the medaka *ψhoxa2b* UER(K20-RE5) but retention of all sequence elements 5’ to this site, including Krox20, BoxA, RE4, RE3 and RE2 (Construct #12), yielded similar results to Construct #8 with a high percentage of transient transgenic embryos showing *eGFP* expression in the hindbrain (80%) and the PAs (80%) (Table 2) and stable-line transgenic embryos generated with Construct #12, showed a similar *eGFP* expression pattern relative to stable-line transgenic embryos generated with Constructs #8, 9 and 10 (Figure 7J–L). Interestingly, similar to the medaka *hoxa2a* UER(K20-RE5) analysis with Construct #6 in which the RE2 and RE5 sequences were eliminated a low percentage of transient transgenic embryos generated with Construct #13, which contained the same deletions, showed *eGFP* expression in the hindbrain (14%) and pharyngeal arches (23%) (Table 2). Further, in comparison to embryos generated with Constructs #8, 9, 10 and 12, *eGFP* expression in embryos generated with Construct #13 was significantly reduced and barely visible (data not shown). Unfortunately, we did not obtain any stable-line transgenic embryos generated with Construct #13 (Data not shown). Overall, these results showed that the sequence containing the elements corresponding to RE3 and RE2 of the medaka *ψhoxa2b* UER(K20-RE5) is required for r3–7 and CNCC expression in PA2 and the posterior PAs but the surrounding elements corresponding to Krox20, BoxA, RE4 and RE5 are not.

**Figure 8:**
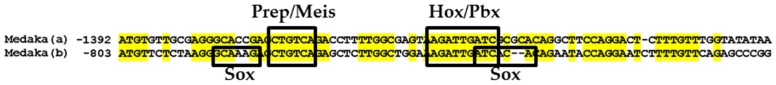
Sequence alignment of the medaka *hoxa2a* r4/CNCC and the *ψhoxa2b* r3–7/CNCC specifying elements. The sequence corresponding to the medaka *hoxa2a* r4/CNCC specifying element is denoted by “a” in parentheses. The sequence corresponding to the medaka *ψhoxa2b* r3–7/CNCC specifying element is denoted by “b” in parentheses. Numbers correspond to genomic base pair positions relative to the ATG start site of *hoxa2a* and *ψhoxa2b*. Base pairs colored in yellow correspond to complete conservation at particular sites across both sequences examined. Black boxed regions correspond to transcription factor binding sites identified in this study.

A comparative genomic sequence analysis revealed that the 88 bp DNA sequence fragment of the medaka *ψhoxa2b* UER(K20-RE5), hereafter referred to as the r3–7/CNCC-specifying element, like the paralogous *hoxa2a* r4/CNCC-specifying element and orthologous *Hoxa2* UER(K20-RE5) genomic sequences of other gnathostomes contains conserved Hox/Pbx, and Prep/Meis transcription factor binding sites (see Figure 5). While these sites may be involved in directing *eGFP* expression in r4 and the CNCCs, their activity may also be redirected to a much broader domain of expression, including r3, r5, r6 and r7. We observed 33 bp differences between the paralogous core sequences upstream of medaka *hoxa2a* and *ψhoxa2b* (Figure 8), and it is possible that the sequence differences between them have allowed the *ψhoxa2b* sequence, but not the *hoxa2a* sequence, to be responsive to transcription factors that mediate expression within r3, r5, r6 and r7 of the hindbrain. An analysis of these sequences in the JASPAR software program showed the presence of several Sox binding elements within the *ψhoxa2b* fragment but not for *hoxa2a*. Sox proteins have been shown to be integral factors in directing *Hox* gene expression among different rhombomeres of the hindbrain [59], indicative that the sequence differences leading to the evolution of additional Sox binding elements in the UER(K20-RE5) of *ψhoxa2b* fragment relative to the *hoxa2a* UER(K20-RE5) may be responsible for the broadened rhombomeric expression observed for the *ψhoxa2b* UER(K20-RE5) relative to the *hoxa2a* UER(K20-RE5).

## 4. Discussion

### 4.1. The Use of Medaka in Reporter Gene Expression Analyses

The *Tol2* transposon system has been used for the generation of transgenic vertebrate lines for several osteichthyans, including zebrafish, *Xenopus*, chicken and mouse [70]. In this study, we showed that the *Tol2* transposon system can be used for both transient and stable-line transgenic analyses of genomic enhancer regions in the Japanese medaka. Although the *Tol2* element is found within the genome of medaka, our control experiments using constructs containing the *Xenopus* eFI-*α*S promoter upstream of *eGFP* showed that this system works similarly in medaka to that of other osteichthyans. Specifically, medaka zygotes that were co-microinjected with constructs containing the *Xenopus* eFI-*α*S promoter and transposon mRNA showed strong reporter gene expression throughout the body whereas embryos that were microinjected solely with constructs containing the *Xenopus* eFI-*α*S promoter were not able to direct reporter gene expression.

Our reporter gene expression results showed that the UER(K20-RE5)s from medaka *hoxa2a* and *ψhoxa2b* are functional, but that they have diverged from one another in their capacity to direct gene expression in the medaka hindbrain and pharyngeal arches. The medaka *hoxa2a* UER(K20-RE5)-directed reporter gene expression in r4 of the hindbrain and the CNCCs of PA2-7 while the medaka *ψhoxa2b* UER(K20-RE5) directed reporter gene expression in r3–7 of the hindbrain and PA2-7. These reporter gene expression results underscore the importance of using a homologous model system for analyzing *cis*-regulatory element control of gene expression during embryonic development. In support, neither the medaka *hoxa2a* nor *ψhoxa2b* UER(K20-RE5)s yielded similar reporter gene expression results when tested in chicken embryos [21]. Specifically, while the *hoxa2a* UER(K20-RE5) did not direct any reporter gene expression in the chicken embryonic head, the *ψhoxa2b* UER(K20-RE5) only directed expression in r3 and r5 but not the CNCCs [21]. The inconsistent results for the medaka *hoxa2a* and *ψhoxa2b* UER(K20-RE5)s within the medaka and chicken embryos suggest that these enhancer regions were not efficient in utilizing the *trans-*acting factors that were present in the heterologous chick model system. In support of this hypothesis, previous reporter gene experiments showed that the mouse *Hoxa2* UER was able to generate reporter gene expression in r3, r5 and the CNCCs in both mouse and zebrafish embryos but unable to direct any reporter gene expression in the hindbrain or CNCCs of chick embryos [29,32]. Similar results were also observed for the fugu *hoxa2a* UER, such that it was shown to be functional when it was tested in both mouse and zebrafish embryos, but not in the chicken [29,32]. Conversely, the UER of chick *Hoxa2* was unable to direct reporter gene expression in either mouse or zebrafish embryos [29]. These results along with our reporter gene expression results of the medaka *hoxa2a* and *ψhoxa2b* UER(K20-RE5)s in medaka embryos suggest that either the functional nature of the sequence elements of the UER, the transcription factors of the genetic regulatory networks that interact with these sequences or a combination of both have diverged greatly among chicken, and possibly other archosaurs, from evolutionarily divergent osteichthyans. Future analyses of the medaka *hoxa2a* and *ψhoxa2b* UER(K20-RE5)s should incorporate the use of zebrafish and/or mouse embryos to assay for species-specific differences in regulatory requirements among these species. Further, orthologous sequences from the mouse *Hoxa2* and zebrafish *hoxa2b* UERs should be tested within medaka embryos.

### 4.2. Medaka Hoxa2a-Directed Gene Expression in the Hindbrain

In this study we showed, using reporter gene assays and whole-mount *in situ* hybridization, that the medaka *hoxa2a* UER(K20-RE5) does not direct expression in r3 and r5 of the hindbrain as expected based on previous whole-mount *in situ* hybridization results of medaka *hoxa2a* [12], but instead directs expression in r4. We found these results interesting since this region of genomic DNA was shown to be conserved structurally with orthologous genomic regions in evolutionarily divergent osteichthyans [21,29,32,59,86]. Further, the *Hoxa2* UERs of zebrafish, mouse and chicken have been shown to direct reporter gene expression in r3 and r5 when tested in their respective homologous host systems [21,25,28,29,30,31,32,59]. An explanation for the lack of reporter gene expression in r3 and r5 in this study is that the genomic DNA sequence tested requires the cooperation of other surrounding sequences within the medaka *hoxa3a-a2a* intergenic region. Our reporter gene analysis of the medaka *hoxa2a* UER(K20-RE5) utilized a 531 bp genomic DNA sequence and contained all of the *cis-*regulatory elements that were tested in the chick embryonic model system [21], including Krox20 and BoxA binding sequences and sequence elements that pertain to RE4, RE3, RE2 and RE5. Functional genomic analyses of the mouse *Hoxa2* UER included regulatory elements orthologous to those mentioned above as well as a RE1 sequence element located upstream of Krox20 [28]. The deletion of the sequences upstream of the Krox20 elements, including the RE1 element, from the mouse *Hoxa2* UER resulted in the loss of reporter gene expression in r3 and r5 of stable-line transgenic mouse embryos [28]. Recent comparative and functional genomic sequence analyses utilizing the UERs of fugu *hoxa2a* and *a2b* and zebrafish *hoxa2b* have shown the presence of a RE1 sequence that is upstream of the Krox20 site in teleosts [29]. Further, these sequences were shown to be much farther upstream from Krox20 in teleosts in comparison to tetrapods [29]. For instance, in comparison to the mouse *Hoxa2* UER, in which the RE1 sequence begins 88 bp upstream of the Krox20 binding site, the orthologous RE1 sequence of medaka *hoxa2a* begins 290 bp upstream of Krox20 [29]. Our analysis of the medaka *hoxa2a* UER(K20-RE5) utilized a sequence that began at genomic bp position −1778, and it excluded the recently identified teleost RE1 sequence that begins at genomic bp postion −2041 [29]. Future reporter gene analyses in medaka embryos should be performed that utilize constructs containing medaka *hoxa3a-a2a* sequences that are orthologous to the “full-length” UERs tested in other gnathostomes to determine if the flanking sequences, including the RE1 element, function in conjunction with the *hoxa2a* UER(Krox20-RE5) sequence to direct reporter gene expression in r3 and r5.

Our reporter gene expression and whole-mount *in situ* hybridization analyses are the first to show the presence of sequences located in the vertebrate *Hoxa3-a2* intergenic regions that are involved in directing reporter gene expression in r4. The *eGFP* expression in r4 of medaka was authenticated using antisense *eGFP* riboprobes and antisense ribpoprobes of specific rhombomeric molecular markers. These molecular markers included medaka *hoxb1a*, which is expressed exclusively in r4 of the hindbrain [16], and medaka *hoxd3a*, which is expressed in r6, r7 and r8 [13]. Microinjection of a series of nested deletion constructs derived from the 531 bp fragment encompassing the medaka *hoxa2a* UER(K20-RE5) showed that the r4-specifying sequence lies within a 89 bp fragment that spans from genomic bp positions −1392 to −1303. Comparative genomic sequence analyses showed the presence of putative transcription factor binding sites, Prep/Meis and Hox/Pbx, located within this fragment. Further, these sites were shown to be highly conserved in *Hoxa2* genomic sequences across several evolutionarily divergent gnathostome vertebrates, including shark, bichir, coelacanth, teleosts and tetrapods (see Figure 5). Interestingly, Hox/Pbx and Prep/Meis binding sites have been shown in multiple studies to be crucial sequences in directing *Hox* expression in r4 [21,33,37]. Future analyses using gel-shift, chromatin immunoprecipitation (ChIP) and site-directed mutagenesis assays must be performed to determine if the Hox/Pbx and Prep/Meis sites within this fragment of the medaka *hoxa2a* UER(K20-RE5) are functional and responsible for directing medaka *hoxa2a* in r4. Further, reporter gene analyses must be performed to determine if the orthologous regions across evolutionarily divergent vertebrate *Hoxa3-a2* intergenic sequences direct reporter gene expression in r4.

A potential explanation for the presence of the r4-directed expression is that the surrounding genomic sequences that were not included in this analysis, such as the RE1 sequence, may function to restrict the r4/CNCC-specifying element from potentiating r4-specific expression or redirect it to direct expression in r3 and r5. In support, recent analyses of CREs responsible for directing fugu *pax2* paralogs, *pax2.1* and *pax2.2*, have shown that the functional activity of the paralogous CREs cognate to these two genes are influenced by interaction among clustered *cis-*regulatory elements in a manner very similar to those from the medaka *hoxa2a* UER(K20-RE5) and their immediately flanking sequences [87]. Specifically, the conserved noncoding element (CNE) pair 1, along with 50 bp extending both 5’ and 3’ of this element for *pax2.1* was shown to direct reporter gene expression in the notochord, muscle and fins [87]. However, when the flanking sequences were removed, the deletion construct containing the more tightly defined CNE pair 1 of fugu *pax2.1* directed an expanded expression pattern that included the otic vesicle, blood, heart, skin and pronephric region [87]. By contrast, the removal of flanking sequences from the paralogous element of *pax2.2* resulted in the gain of directed reporter gene expression in the fins but loss of expression from the eye and otic vesicle [87]. By comparison, we argue that the inclusion of flanking sequences to the medaka *hoxa2a* UER(K20-RE5), including the RE1 sequence, may change the functional activity of the medaka UER(K20-RE5) region to direct reporter gene expression in r3 and r5 but negate expression in r4.

### 4.3. Hoxa2a-Directed Gene Expression in the Cranial Neural Crest Cells

Beyond the hindbrain expression directed activity of the medaka *hoxa2a* UER(K20-RE5), this genomic DNA region was also shown to possess functional activity that directs *hoxa2a* expression in the CNCCs. Our whole-mount *in situ* hybridization results from stable-line transgenic medaka embryos have shown that the medaka *hoxa2a* UER(K20-RE5) directs reporter gene expression in the migratory CNCCs and is involved in maintaining *hoxa2a* expression in post-migratory CNCCs in PA2 and the posterior arches up until the chondrogenic stages of craniofacial skeleton development. Several functional genetic studies have shown that *Hox* PG2 gene expression that persists late into post-migratory CNCC stages of development is necessary for the proper patterning of the craniofacial skeleton [45,56,62,64,88]. Our analyses suggest that the genomic sequences of the medaka UER(K20-RE5) are utilized *in vivo* to maintain medaka *hoxa2a* expression up until chondrogenesis in PA2 and the posterior arches to allow for proper craniofacial skeleton development.

Although we observed *eGFP* expression patterns in the post-migratory CNCCs that were directed by the medaka *hoxa2a* UER(K20-RE5), they did not fully phenocopy the post-migratory CNCC expression patterns shown by medaka *hoxa2a* whole-mount *in situ* hybridization experiments [12]. For instance, we observed very low levels of *eGFP* expression within PA2 but strong *eGFP* expression in the posterior arches in stable-line transgenic medaka embryos generated with Constructs #1, 2 and 5 (see Figure 4). However, we observed strong *eGFP* expression in PA2 and the posterior arches in stable-line transgenic embryos generated with Construct #7 (see Figure 4). Thus, our reporter gene expression results suggest that the genomic sequences analyzed in this study, as well as other surrounding sequences, are necessary for medaka *hoxa2a* to be expressed and ultimately function in patterning the jaw support elements that are derived from PA2 and the pharyngeal jaw apparatus from the posterior pharyngeal arches. Future functional genomic studies using the medaka *hoxa2a* UER(K20-RE5) and flanking genomic sequences will be required to fully phenocopy the medaka *hoxa2a* expression pattern in the post-migratory CNCCs.

Our functional and comparative genomic analyses showed that the 89-bp region (Construct #7) responsible for directing reporter gene expression in the CNCCs of PA2 and the posterior arches contains Hox/Pbx and Prep/Meis binding sites (see Figure 5). These results are suggestive of auto- and/or cross-regulation of medaka *Hox* PG2 genes in the PAs. Recent functional genomic analyses in mouse showed that Hox/Pbx and Meis binding sequences located within the proximal promoter region upstream of mouse *Hoxa2* are necessary for *Hoxa2* expression and function in PA2 and posterior arches [24]. Further, this study showed that mouse *Hoxa2*, *Pbx* and *Meis* utilize these binding sites to function in a positive feedback loop that maintains and amplifies *Hoxa2* expression in the PAs [24]. Specifically, *Hoxa2* was shown to positively regulate *Meis* within the PAs, and *Meis* along with *Hoxa2*, were shown to work in concert in amplifying *Hoxa2* expression within these domains [24]. Unlike tetrapods, many species of ray-finned fishes contain multiple *Hox* PG2 genes that are expressed in PA2 and the posterior arches up until chondrogenesis, including *hoxa2a*, *a2b* and *b2a* [12,14,19,56,89]. Further, functional and comparative genomic sequence analyses have shown that Hox/Pbx and Prep/Meis sites are located within the *hoxb3a-b2a* intergenic region of ray-finned fishes and *Hoxb3-b2* of lobe-finned fishes [37,89]. These results suggest that the *Hox*/*Pbx* and *Prep*/*Meis* sites are used by teleost *Hox* PG2 genes in auto- and cross-regulatory mechanisms of gene expression in the PAs. In support, the independent knockdown of *hoxa2a* and *b2a* in tilapia resulted in altered expression levels of tilapia *Hox* PG2 genes in the PAs [64]. Specifically, the knockdown of tilapia *hoxa2a* resulted in a reduction of *hoxa2a* expression from PA2 and the posterior arches and *hoxb2a* expression from PA2, and the knockdown of *hoxb2a* resulted in the reduction of *hoxa2a* and *a2b* expression from PA2 [64]. These altered expression levels corresponded to the varying severity of loss-of-function phenotypes in tilapia, wherein the loss of *hoxa2a* resulted in a full homeotic transformation the PA2-derived skeletal elements but the loss of *hoxb2a* resulted in altered bony morphology of PA2 elements without a full homeotic transformation [64]. By contrast, in zebrafish the knockdown of both *hoxa2b* and *b2a* were required to obtain a full homeotic transformation of PA2-derived skeletal elements [56], which suggests that both zebrafish *Hox* PG2 genes function redundantly in utilizing the Hox/Pbx and Prep/Meis binding sites to auto- and cross-regulate each other’s expression in PA2. Future functional genetic analyses must be performed to understand if medaka *hoxa2a* and *b2a* utilize the Hox/Pbx and Prep/Meis binding sites similar to *hoxa2b* and *b2a* of zebrafish or *hoxa2a* and *b2a* of tilapia.

### 4.4. Functional Nature of the Medaka ψHoxa2b r3/5 Enhancer Region

Our results from transient and stable-line transgenesis and whole-mount *in situ* hybridization analyses using anti-*eGFP* riboprobes have shown that the medaka *ψhoxa2b* UER(K20-RE5) is functional and is able to direct robust reporter gene expression in r3–7 of the hindbrain, PA2 and the posterior PAs. These results were contrary to our expectations based on whole-mount *in situ* hybridization analyses of medaka *ψhoxa2b*, which showed that this pseudogene is expressed strongly in noncanonical *Hox* PG2 expression domains but not at all in the characteristic hindbrain and CNCC compartments [12]. These contradictory results suggest that regions of genomic sequences surrounding the medaka *ψhoxa2b* UER(K20-RE5) function to redirect the activity of this region to mediate expression in the noncanonical *Hox* PG2 embryonic compartments. In addition to the unexpected functional nature of the medaka *ψhoxa2b* UER(K20-RE5), this region was observed to be divergent in function from the UER(K20-RE5) of medaka *hoxa2a*, which directs gene expression in r4, PA2 and the posterior arches. Further, deletion mutagenesis of the medaka *ψhoxa2b* UER(K20-RE5) showed that a relatively short sequence that is paralagous to the r4/CNCC-specifying element of the medaka *hoxa2a* UER(K20-RE5) was responsible for directing reporter gene expression in r3–7, PA2 and the posterior PAs. Comparative genomic analysis of the medaka *ψhoxa2b* r3–7/CNCC-specifying element revealed the presence of conserved Hox/Pbx and Prep/Meis binding sites (see Figure 5). However, this sequence was shown to be divergent from the medaka *hoxa2a* r4/CNCC-specifying element by 33 bp (see Figure 8). It is possible that these substitutions between the medaka *hoxa2a* and *ψhoxa2b* genomic sequences have allowed the *ψhoxa2b-*specific sequence, but not the *hoxa2a*, to be receptive to transcription factors expressed within r3, r5, r6 and r7 of the hindbrain. In support, analyses of the CREs that direct fugu *pax2.1* and *pax2.2* gene expression showed that while there is high sequence similarity between several paralogous CREs, these elements direct divergent expression patterns [87]. For instance, although the pair 1 CNEs of *pax2.1* and *pax2.2* show roughly 90% similarity in sequence, the pair 1 CNE of fugu *pax2.1* directs gene expression in the spinal cord, muscle, notochord and fins while the paralogous sequence of *pax2.2* directs expression in the forebrain, midbrain, hindbrain, spinal cord, eye, otic vesicle, blood, heart, skin, thyroid region and pronephric region [87]. Interestingly, our analyses of the medaka *Hox cis-*regulatory sequences using the JASPAR software program revealed several Sox binding elements within the *ψhoxa2b* r3–7/CNCC-specifying element but not in the *hoxa2a* r4/CNCC-specifying element (see Figure 8). Sox proteins have been shown to be involved in directing *Hox* gene expression in several rhombomeres of the hindbrain [59], so it is possible that these elements were involved in directing reporter gene expression in r3, r5, r6 and r7. The base pair mutations between the r3–7/CNCC-specifying element of medaka *ψhoxa2b* and the paralogous r4/CNCC-specifying element may have occurred due to relaxation of selective pressures on the *ψhoxa2b* genomic sequence after the inactivation of the *hoxa2b* gene in the lineage leading to medaka [12].

Our reporter gene expression results of the medaka *ψhoxa2b* UER(K20-RE5) coupled with previous expression pattern results of medaka *ψhoxa2b* during embryonic development may provide an excellent example of how evolutionary changes in noncoding sequences contribute to morphological evolution. An increasing body of evidence in the field of evolutionary and developmental biology has shown that sequence changes in conserved noncoding sequences can affect expression patterns of developmentally important genes, and the co-option of such genes in divergent embryonic domains can lead to morphological novelties (see [90]). Assuming that the medaka *ψhoxa2b* transcript gives rise to a functional product that can affect developmental specification, the heterotopic shift in expression that lead to medaka *ψhoxa2b* co-option into noncanonical *Hox* PG2 developmental compartments may have contributed to unique morphological and functional traits. Based on *in silico* translation of the likely mRNA product derived from *ψhoxa2b* cDNA clones, it is clear that this pseudogene cannot generate a fully functional Hox protein product [12]. However, it can generate a truncated protein that possesses a hexapeptide that is conserved with orthologous hexapeptides of *hoxa2b* genes from evolutionarily divergent teleosts [12]. Since the hexapeptide of *Hox* genes are known to mediate interactions with other transcription factors, especially *Pbx*, there is the tantalizing possibility that the truncated product of *ψhoxa2b* translation could interact with *Pbx* and influence transcriptional activity in developmental compartments in which it is expressed. In the case of medaka, which is a member of the beloniform fishes (an order that includes the flying fishes), these mechanisms may be responsible for generating divergent morphological characters specific to beloniforms. Of particular interest in this regard is the fact that we have documented expression of *ψhoxa2b* in the developing pectoral fin buds [12]. Several functional genetic studies have shown that *Meis*, *Pbx*, and posterior *Hox* genes within the A and D clusters function to pattern the proximal-distal axis of the developing limbs [91,92,93,94,95,96]. Likewise, *Pbx* has been shown to be involved in the upregulation of posterior *HoxA* and *D* genes, and that the loss of function of either *Pbx* or posterior *HoxA* and *D* cluster genes results in truncated limbs [92,93]. Given the location of expression of the pseudogene and its potential to interact with *Hox* gene products in the fin bud progress zone, it is possible that the co-option of *ψhoxa2b* expression into this compartment may have influenced the evolution of elongated pectoral fins in the lineage leading to flying fish, a remarkable adaptation that is restricted to this taxonomic clade. In order to test these hypotheses, functional genomic analyses of the *hoxa2b* UER(K20-RE5)) of other beloniform fishes must be tested within the medaka. Further, phylogenetic analyses must be performed in order to understand if the *hoxa2b* inactivation is specific to the lineage leading to medaka or if it occurred earlier in the beloniform radiation to include other members of this taxonomic clade.

## 5. Conclusions

In conclusion, our reporter gene analyses showed that the medaka *hoxa2a* and *ψhoxa2b* UER(K20-RE5)s are functionally divergent from one another. While both UER(K20-RE5)s were shown to direct reporter gene expression in the CNCCs of PA2 and the posterior PAs, the medaka *hoxa2a* UER(K20-RE5) directed expression in r4 of the hindbrain while the medaka *ψhoxa2b* directed expression in r3–7. These results are different from heterologous reporter gene expression results of the medaka *hoxa2a* and *ψhoxa2b* UER(K20-RE5)s when they were tested in chicken embryos. In chicken, the medaka *hoxa2a* UER(K20-RE5) did not direct expression in the hindbrain or pharyngeal arches while the *ψhoxa2b* UER(K20-RE5) directed reporter gene expression in just r3 and r5 of the hindbrain. Overall, our analyses show that when tested independent of their natural genomic surroundings, the UER(K20-RE5)s of medaka *hoxa2a* and *ψhoxa2b* are able to direct expression within r4 and *Hox* PG2 canonical domains, respectively.

## Figures and Tables

**Table 1:** Primers used for the amplification of the medaka *hoxa2a* and *ψhoxa2b* UER(K20-RE5)s and the exclusion of sequence elements for the functional testing of these regulatory regions. The 5′-start site for primers pertains to the nucleotide base position with respect to the ATG start site of the downstream gene (*hoxa2a*) or pseudogene (*ψhoxa2b*).

Primer	Sequence 5′ to 3′	5′ Start Site
Medaka *hoxa2a* Genomic Primers		
A2a For	TTATTCCCACAACCCTTTCATTTCG	−2691
A2a Rev	CACACTCAGCCACAATCTCTTCTTC	1846
Medaka *ψhoxa2b* Genomic Primers		
A2b For	ACACAGCAGGGGTCAACAATAGGTC	−3093
A2b Rev	ATAGGCAGAGCACGAAAACAAAATG	3193
Medaka *hoxa2a* UER(K20-RE5) Forward Primers		
AF1	GATCGATATCGAACAGGCTGAAATCCACTGAATGC	−1778
AF2	GATCGATATCGCTTCTAATCTGAGAAGCCAGTGTTTC	−1468
AF3	GATCGATATCATGTGTTGCGAGGGCACCGAGCTGTC	−1392
AF4	GATCGATATCGAGTAAGATTGATCGCGCACAGGCTTC	−1354
Medaka *hoxa2a* UER(K20-RE5) Reverse Primers		
AR1	GATCGAATTCGTTTGCTGTGGAACAGAGGAAAGAAG	−1247
AR2	GATCGAATTCTTATATACCAAACAAAGAGTCCTGG	−1303
AR3	GATCGAATTCTTACTCGCCAAAAGGTCTGACAGCTC	−1348
Medaka *ψhoxa2b* UER(K20-RE5) Forward Primers		
BF1	GATCGATATCATGTGCCAACACCCACTCACCCCAG	−1068
BF2	GATCGATATCCTTCGCTCCGCACCGAGGGCATCCTC	−868
BF3	GATCGATATCATGTTCTCTAAGGGCAAAGAGCTGTC	−803
BF4	GATCGATATCTGGAAAGATTGATCACACAGAATACC	−765
Medaka *ψhoxa2b* UER(K20-RE5) Reverse Primers		
BR1	GATCGAATTCAAAAAGCTGCAGGAAAAGGAGGGGATC	−671
BR2	GATCGAATTCCCGGGCTCTGAACAAAAGATTCCTG	−715
BR3	GATCGAATTCTTTCCAGCCAAGAGCTCTGACAGCTC	−759

**Table 2:** Primer pairs used for the amplification of sequences of the medaka *hoxa2a* and *ψhoxa2b* UER(K20-RE5)s used in functional genomic analyses. Schematics of constructs, frequencies of transient transgenic embryos (F0s) showing reporter gene expression in the hindbrain and CNCCs, and numbers of stable-line transgenic adults (F1s) are shown. K, Krox20, B, BoxA, R, Rhombomeric element, *, embryos showing nearly undetectable *eGFP* expression in the hindbrain and pharyngeal arches.

Construct	Primer Pairs	Amplicon Length	Construct Schematic	Hindbrain Expression (F0)	CNCC Expression (F0))	F1s
Medaka *hoxa2a* UER(K20-RE5) construct design and transgenic analysis						
1	AF1/AR1	531 bp	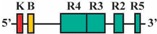	42/48 (87.5%)	42/48 (87.5%)	3
2	AF2/AR1	221 bp	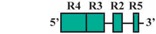	64/84 (76%)	56/84 (67%)	3
3	AF3/AR1	145 bp	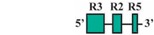	39/49 (80%)	41/49 (84%)	0
4	AF4/AR1	107 bp	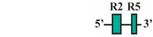	7/47 (15%) *	23/49 (49%) *	0
5	AF1/AR2	475 bp	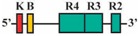	42/50 (84%)	42/50 (84%)	4
6	AF1/AR3	430 bp	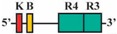	0/52 (0%)	0/52 (0%)	0
7	AF3/AR2	89 bp	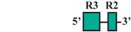	52/62 (84%)	52/62 (84%)	4
Medaka *ψhoxa2b* UER(K20-RE5) construct design and transgenic analysis						
8	BF1/BR1	397 bp	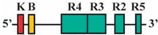	46/51 (90%)	46/51 (90%)	2
9	BF2/BR1	197 bp	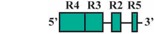	47/52 (90%)	47/52 (90%)	4
10	BF3/BR1	132 bp	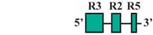	33/38 (87%)	33/38 (87%)	3
11	BF4/BR1	94 bp	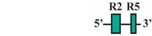	27/52 (52%) *	27/52 (52%)*	0
12	BF1/BR2	353 bp	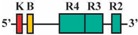	33/41 (80%)	33/41 (80%)	3
13	BF1/BR3	309 bp	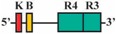	9/64 (14%) *	15/64 (23%) *	0

## Abbreviations

The following abbreviations are used in this manuscript:

A-P: anterior-posterior
bp: base pair
CNCC: cranial neural crest cell
CNE: conserved noncoding element
CRE: *cis-*regulatory element
eGFP: enhanced green fluorescent protein
ERM: embryo rearing medium
*E. coli*: *Escherichia coli*
GFP: green fluorescent protein
NC: neural crest
PA: pharyngeal arch
PG: paralog group
r: rhombomere
RE: rhombomeric element
UER: upstream enhancer region
UER(K20-RE5): upstream enhancer region spanning 5′ from Krox20 binding element to the 3′ RE5 element
